# Application of Multiscale Entropy in Mechanical Fault Diagnosis of High Voltage Circuit Breaker

**DOI:** 10.3390/e20050325

**Published:** 2018-04-28

**Authors:** Longjiang Dou, Shuting Wan, Changgeng Zhan

**Affiliations:** 1 Department of Mechanical Engineering, North China Electric Power University, Baoding 071003, China; 2 Hangzhou Steam Turbine Co., Ltd., Hangzhou 310000, China

**Keywords:** vibration signal, multiscale entropy, support vector machine, fault diagnosis

## Abstract

Mechanical fault diagnosis of a circuit breaker can help improve the reliability of power systems. Therefore, a new method based on multiscale entropy (MSE) and the support vector machine (SVM) is proposed to diagnose the fault in high voltage circuit breakers. First, Variational Mode Decomposition (VMD) is used to process the high voltage circuit breaker’s vibration signals, and the reconstructed signal can eliminate the effect of noise. Second, the multiscale entropy of the reconstructed signal is calculated and selected as a feature vector. Finally, based on the feature vector, the fault identification and classification are realized by SVM. The feature vector constructed by multiscale entropy is compared with other feature vectors to illustrate the superiority of the proposed method. Through experimentation on a 35 kV SF_6_ circuit breaker, the feasibility and applicability of the proposed method for fault diagnosis are verified.

## 1. Introduction

High voltage circuit breakers are important components used for protection and control purposes in power systems. The reliability of the breakers will affect the security and stability of a power system, which means the maintenance of the breakers is one of the important daily tasks of an electrical power department. To get the operating characteristics of a circuit breaker, preventive test methods are adopted during maintenance. Such methods are not only time-consuming but also in frequent operation, and excessive maintenance will reduce the reliability of a circuit breaker [1,2]. Therefore, fault diagnosis of a circuit breaker is becoming more important. Through research, one can find potential problems in advancing and enhancing the reliability of power systems. The related research has great significance.

The vibration signal and acoustic signal of a high voltage circuit breaker contain abundant information. For example, the state of the circuit breaker, such as loose screws and iron core jamming, can be found by analyzing these signals [3,4,5]. Therefore, fault diagnosis of a circuit breaker has become a major research topic [6,7,8,9,10,11,12,13,14]. Runde et al. [15] used acoustic signals to assess the condition of circuit breakers first. Also, they verified the efficiency of the diagnostic technique. Similarly, Hussain et al. [16] explored an expert system for acoustic diagnosis of circuit breakers, which improved the development of the acoustic diagnosis technology in circuit breakers. Mei et al. [17] proposed a fault diagnosis method based on particle-swarm fused kernel fuzzy C-means and support vector machine (SVM). Zhang et al. [18] proposed an extraction method based on ensemble empirical mode decomposition (EEMD) and energy entropy. Classification results showed that the diagnosis approach can identify the fault patterns effectively. Cheng et al. [19] adopted the factor analysis to optimize and reduce the dimensions of characteristic parameters and then classified different states of a circuit breaker using SVM, which is improved by particle-swarm optimization. By combining wavelet packets with characteristic entropy, Sun et al. [20] proposed a new method for diagnosing a circuit breaker’s fault. Wu et al. [21] applied Empirical Mode Decomposition (EMD) energy to extract the feature vector from the vibration signal of a circuit breaker, and the results showed that the method could identify different vibration signals and fault types. Because the acoustic signal is easily disturbed by the environment, relatively, the vibration signal has more reliable stability than the acoustic signal, so we focus on the vibration signal. Although the methods for processing the vibration signal have successfully illustrated good fault-diagnosis results, there are still some drawbacks. The collected vibration signals are inevitably mixed with electromagnetic and other noises due to the end effect and the mode-mixing problems. Therefore, the result of using EMD and EEMD to eliminate the noise in the vibration signal is not ideal. Furthermore, the characteristics of the proposed feature vectors are not obvious.

In 2014, American researcher Konstantin Dragomiretskiy proposed a new method of signal processing called Variational Mode Decomposition (VMD) [22]. This method gets rid of cyclic-recursive screening to obtain the signal components. Unlike the conventional methods of signal processing, the VMD method adopts a nonrecursive processing strategy with a solid theoretical background. In summary, the VMD has solved the variational problems, making the sum of each mode’s estimated bandwidth minimum and assuming that each mode has limited frequency bandwidth with different center frequency. To solve the variational problems, the method of alternating the direction multiplier can be adopted to continually update the model and its center frequency. Furthermore, each mode will be demodulated into the corresponding base bands gradually. Finally, all the modes and the corresponding center frequencies can be extracted. The decomposition mode offers good stability and can reflect the characteristics of signal singularity. Due to these excellent characteristics, VMD has been applied to signal processing [23,24,25].

In order to solve the above problems, we used VMD to process a circuit breaker’s vibration signals. The signal component that contains the characteristic information can be extracted from the original signal. Then, the multiscale entropy (MSE) is introduced as a feature vector. Finally, the SVM is applied to classify the circuit breaker’s states. The experimental results demonstrate that the proposed method can extract the fault vibration signal’s feature vector quickly and efficiently. In addition, it can classify the circuit breaker’s states perfectly.

This paper is organized as follows. The mathematical model of VMD is introduced in Section 2. Multiscale entropy is presented in Section 3. Fault diagnosis based on SVM is introduced in Section 4. Experimental application is illustrated in Section 5. Some conclusions are given in Section 6.

## 2. Variational Mode Decomposition

### 2.1. Principle of VMD

K. Dragomiretskiy proposed an entirely nonrecursive variational mode decomposition model in 2014 where the modes are extracted concurrently. Its framework is a variable problem and the sum of the estimated bandwidth of each mode can be minimized. First, assume that each mode is a finite bandwidth with different center frequencies. Then, using the alternating direction multiplier method to update the mode and its center frequency constantly, we demodulate the mode to the corresponding baseband gradually. Finally, each mode and corresponding center frequency is extracted together. This method is different from the EMD and LMD algorithms. It highlights the local characteristics of the data and shows better noise robustness.

VMD includes the construction and solution of the variational problem. It involves three important concepts called Wiener filtering, Hilbert transform, and frequency mixing.

#### 2.1.1. Construction of Variational Problem

Assume that each mode is band limited with a center frequency, seek the *k* mode functions *u_k_*(*t*), and minimize the sum of the estimated bandwidth of each mode. The constraint is the sum of the modes equal to the input signal *f*. The specific construction steps are below.

(1) Through Hilbert transform, get the unilateral frequency spectrum of each mode *u_k_*(*t*).
(2) After mixing with an exponential tuned to the respective estimated center frequency, shift the mode’s frequency spectrum to the “baseband”.
(3) Compute the squared *L*^2^-norm of the above demodulated signal gradient. The bandwidth of each modal signal is estimated. The resulting constrained variational problem is below:
  (1) $$\{\begin{array}{c} \underset{\left\{u_{k}\right\},\left\{w_{k}\right\}}{\min}\left\{\sum_{k}{\left\|\partial_{t}\left[\left(\delta \left(t\right)+\frac{j}{\pi t}\right)*u_{k}\left(t\right)\right]e^{-j\omega_{k}t}\right\|}_{2}^{2}\right\}\\ s.t.\sum_{k}u_{k}=f \end{array}$$
  where $\left\{u_{k}\right\}:=\left\{u_{1},\ldots ,u_{k}\right\}$ and $\left\{\omega_{k}\right\}:=\left\{\omega_{1},\ldots ,\omega_{k}\right\}$ are shorthand notations for the set of all modes and their center frequencies.

#### 2.1.2. Solution of Variational Problem

(1) In order to render the problem unconstrained, we make use of both a quadratic penalty term α and Lagrangian multipliers λ(t). The quadratic penalty is a classic way to encourage reconstruction fidelity typically in the presence of additive Gaussian noise. Lagrangian multipliers are a common way of enforcing constraints strictly. We introduce the augmented Lagrangian using the equation below:
  (2) $$L\left(\left\{u_{k}\right\},\left\{\omega_{k}\right\},\lambda \right)=\alpha {\sum_{k}\left\|\partial_{t}\left[\left(\delta \left(t\right)+\frac{j}{\pi t}\right)*u_{k}\left(t\right)\right]e^{-j\omega_{k}t}\right\|}_{2}^{2}+{\left\|f\left(t\right)-\sum_{k}u_{k}\left(t\right)\right\|}_{2}^{2}+\langle \lambda \left(t\right),f\left(t\right)-\sum_{k}u_{k}\left(t\right)\rangle$$
(2) The solution to the original minimization problem is found to be the saddle point of the augmented Lagrangian in a sequence of iterative suboptimizations called alternate direction method of multipliers (ADMM) [19]. $u_{k}^{n+1}$, $\omega_{k}^{n+1}$, $\lambda_{k}^{n+1}$ are alternately updated using the ADMM approach. The updates of $u_{k}^{n+1}$, …, $\lambda_{k}^{n+1}$ are below:
  (3) $${\hat{u}}_{k}^{n+1}\left(\omega \right)=\frac{\hat{f}\left(\omega \right)-\sum_{i\neq k}{\hat{u}}_{i}\left(\omega \right)+\frac{\hat{\lambda }\left(\omega \right)}{2}}{1+2\alpha {\left(\omega -\omega_{k}\right)}^{2}}$$
  (4) $$\omega_{k}^{n+1}=\underset{\omega_{k}}{\arg\min}\left\{\int_{0}^{\infty }{\left(\omega -\omega_{k}\right)}^{2}{\left|{\hat{u}}_{k}\left(\omega \right)\right|}^{2}d\omega \right\}$$
  This quadratic problem is solved in Equation (5):
  (5) $$\omega_{k}^{n+1}=\frac{\int_{0}^{\infty }\omega {\left|{\hat{u}}_{k}\left(\omega \right)\right|}^{2}d\omega }{\int_{0}^{\infty }{\left|{\hat{u}}_{k}\left(\omega \right)\right|}^{2}d\omega }$$
  where $\omega_{k}^{n+1}$ is the center of gravity of the corresponding mode’s power spectrum and wiener filtering is carried out on $\hat{f}\left(\omega \right)-\sum_{i\neq k}{\hat{u}}_{i}\left(\omega \right)$ to obtain ${\hat{u}}_{k}^{n+1}\left(\omega \right)$.

### 2.2. Algorithm of VMD

(1) Initialize $\left\{{\hat{u}}_{k}^{1}\right\}$, $\left\{\omega_{k}^{1}\right\}$, $\left\{{\hat{\lambda }}^{1}\right\}$, n=0;
(2) According to Equations (3) and (5), update $u_{k}$ and $\omega_{k}$;
(3) Update λ:
  (6) $${\hat{\lambda }}^{n+1}\left(\omega \right)\leftarrow {\hat{\lambda }}^{n}\left(\omega \right)+\tau \left[\hat{f}\left(\omega \right)-\sum_{k}{\hat{u}}_{k}^{n+1}\left(\omega \right)\right]$$
(4) Given the discriminant accuracy e≥0, if ${\sum_{k}\left\|{\hat{u}}_{k}^{n+1}-{\hat{u}}_{k}^{n}\right\|}_{2}^{2}/{\left\|{\hat{u}}_{k}^{n}\right\|}_{2}^{2}<e$, stop the iteration. Otherwise, return to step 2.

The algorithm of VMD is very simple. First, the modes are updated directly in the frequency domain and then transformed to the time domain by using the inverse Fourier. Next, with the center of gravity of each mode power spectrum, the center frequency is re-estimated and updated cyclically.

## 3. Multiscale Entropy

Based on sample entropy, multiscale entropy is equivalent to calculating the sample entropy of the time series at different scales. Multiscale entropy is a nondimensional indicator of signal characteristics and it represents the complexity of the time series on different scales [26].

### 3.1. Sample Entropy

Sample entropy theory was proposed by Richman and Moorman [27], and they defined the calculation process details. Sample entropy is the natural logarithm of the conditional probability and is introduced below.

(1) Assume the original time series {*x*(*i*) } is *x*(1), *x*(2), …, *x*(*N*). The *m* dimensional vector is constructed as follows: *X*(*i*) = [*x*(*i*), *x*(*i*+1), …, *x*(*i* + *m* − 1)], where *i* = 1, 2, …, *N* − *m* + 1.
(2) *d_m_*(*X*(*i*), *X*(*j*)) is the max distance between *X*(*i*) and *X*(*j*):
  (7) $$d_{m}\left(X\left(i\right),X\left(j\right)\right)=\underset{0\sim m-1}{\max}\left|x\left(i+k\right)-x\left(j+k\right)\right|$$
  For each *i*, the max distance between *X*(*i*) and *X*(*j*) (*j* = 1, 2, …, *N* − *m* + 1, *j* ≠ *i*) is calculated, then the *d_m_*(*X*(*i*), *X*(*j*)) is obtained.
(3) *r* is the tolerance for accepting matches (*r* > 0), $B_{i}^{m}(r)$ is the ratio of the number of *d_m_*(*X*(*i*), *X*(*j*)) < *r* to total distance:
  (8) $$B_{i}^{m}(r)=\frac{1}{N-m}num\left\{d_{m}(X(i),X(j))<r\right\}$$
  where *i* = 1, 2, …, *N* − *m* + 1, *j* ≠ *i* and *num* is the number of *d_m_*(*X*(*i*), *X*(*j*)) < *r*. $B_{i}^{m}(r)$ is the matching probability of *X*(*j*) and *X*(*i*).
(4) $B^{m}(r)$ is the average of $B_{i}^{m}(r)$:
  (9) $$B^{m}(r)=\frac{1}{N-m+1}\sum_{i=1}^{N-m+1}B_{i}^{m}(r)$$
(5) m→m+1, $B^{m+1}(r)$ is calculated according to Equations (7) and (9).

The sample entropy is defined below:

(10) $$SampEn\left(N,m,r\right)=-\ln\left[B^{m+1}\left(r\right)/B\left(r\right)\right]$$

### 3.2. Multiscale Entropy

The sample entropy of the signal is calculated in a certain *m* and the multiscale entropy can be obtained in different *m,* which is described below.

(1) By setting the embedded dimension *m* and the similarity tolerance *r*, a new coarse-grained time series is created:
  (11) $$y_{j}^{\tau }=\frac{1}{\tau }\sum_{i=\left(j-1\right)\tau +1}^{j\tau }x_{i}\;1\leq j\leq \frac{N}{\tau }$$
  where *τ* is scale factor, *τ* =1, 2, ….
  For *τ* = 1, *y_j_*(1) is the original time series. For *τ* = 2, 3, …, the original time series is divided into *N*/*τ* coarse-grained time series *y_j_*(*τ*);
(2) We calculate an entropy measure for each coarse-grained time series plotted as a function of the scale factor *τ.* This procedure is called multiscale entropy analysis.
  (12) $$MSE\left(x,\tau ,m,r\right)=SampEn\left(y^{\tau },m,r\right)$$

## 4. Fault Diagnosis Based on SVM

The operating life of a circuit breaker is limited. For example, the operating life of the SIEMENS circuit breaker is 10,000–30,000, which is longer than Chinese circuit breakers. In our experimental test, a screw was loosened after about 700 times, which lead to a change in vibration. In order to ensure the consistency of the vibration signal, we collected limited samples. In the classification algorithm, SVM is suitable for small sample classification. The SVM and fault diagnosis process are introduced as follows.

### 4.1. Principle of SVM 

**Figure 1:**
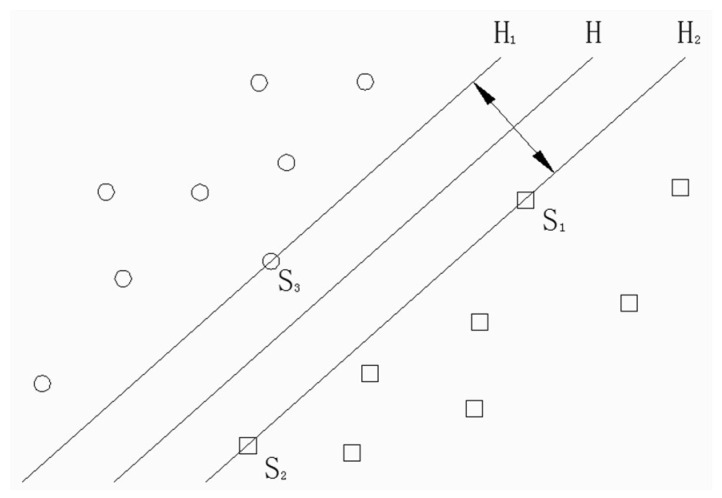
Optimal separating hyperplane.

SVM is a kind of universal learning algorithm which can achieve the minimum structure risk. It uses kernel functions to convert samples mapped to high-dimensional feature space. Then, the optimal separating hyperplane is constructed and the classification interval is made to be the largest. Therefore, the support vector machine is suitable for small sample data classification. The basic idea of SVM is shown in Figure 1.

In Figure 1, circles and squares represent two types of training samples. The two types of training samples are separated by optimal separating hyperplane *H* accurately. Line *H*_1_ and line *H*_2_ are parallel to hyperplane *H*. Additionally, they pass through the samples closest to *H*. The distance between *H*_1_ and *H*_2_ is the classification interval and the samples on *H*_1_ and *H*_2_ are the support vectors. The sum of distance between support vectors and the optimal separating hyperplane is 2||*w*||. Therefore, the problem of constructing an optimal hyperplane is transformed into an optimization problem:

(13) $$\{\begin{array}{c} \underset{w,b}{\min}\frac{1}{2}{\left\|w\right\|}^{2}\\ s.t.\;y_{i}\left(w\cdot x+b\right)\geq 1,i=1,2,\cdot \cdot \cdot ,l \end{array}$$

where w is the normal vector of optimal hyperplane and b is the threshold.

Some samples cannot be classified by the hyperplane correctly due to linear extension. Therefore, we introduced the relaxation vector $\varepsilon_{i}\geq 0$, so that the constraint of hyperplane becomes $y_{i}\left(w\cdot x+b\right)+\varepsilon_{i}\geq 1$. At the same time, the penalty parameter *C* w added to obtain the minimum $\varepsilon_{i}$. The objective function is below.

(14) $$\{\begin{array}{c} \underset{w,b}{\min}\frac{1}{2}{\left\|w\right\|}^{2}+C\sum_{i=1}^{l}\varepsilon_{i}\\ s.t.\;y_{i}\left(w\cdot x+b\right)\geq 1-\varepsilon_{i},i=1,2,\cdot \cdot \cdot ,l \end{array}$$

We used the Lagrangian multiplier to solve the above problem and get the optimized objective function presented below.

(15) $$\max L=\sum_{i=1}^{l}\alpha_{i}\alpha_{j}y_{i}y_{j}x_{i}^{T}x_{j}$$

The corresponding constraints are $\sum_{i=1}^{l}\alpha_{i}y_{i}=0$, $0\leq \alpha_{i}\leq C$. $\alpha_{i}$ is a Lagrangian multiplier. Extended to nonlinear problems, the samples in the low-dimensional space can be mapped into high-dimensional space by using the mapping function ϕ(x). Therefore, the samples are linearly separable in the high-dimensional space. The kernel function is defined as $K\left(x_{i},x_{j}\right)=\phi {\left(x_{i}\right)}^{T}\phi \left(x_{j}\right)$. The optimization objective function is shown below.

(16) $$\max L=\sum_{i=1}^{l}\alpha_{i}-\frac{1}{2}\sum_{i=1,j=1}^{l}\alpha_{i}\alpha_{j}y_{i}y_{j}K\left(x_{i},x_{j}\right)$$

### 4.2. Fault Diagnosis Process

In the proposed extraction method based on VMD and multiscale entropy, the VMD is taken as a preprocessor to decompose the vibration signal into an ensemble of band-limited intrinsic mode functions (IMFs) and the multiscale entropy is used to extract the fault features. The fault diagnosis steps are described below.

(1) the vibration signal into appropriate IMFs by VMD.
(2) Calculate the correlation coefficient between the original vibration signal and its IMFs.
(3) Sort the IMFs from small to large in terms of the correlation coefficients and the largest five IMFs are selected to reconstruct the signal.
(4) Calculate the multiscale entropy of the reconstructed signal according to (7)–(12) and the feature vector is constructed by multiscale entropy.
(5) Fault classification is realized by the SVM based on the feature vector.

## 5. Experimental Application 

### 5.1. Data Acquisition

**Figure 2:**
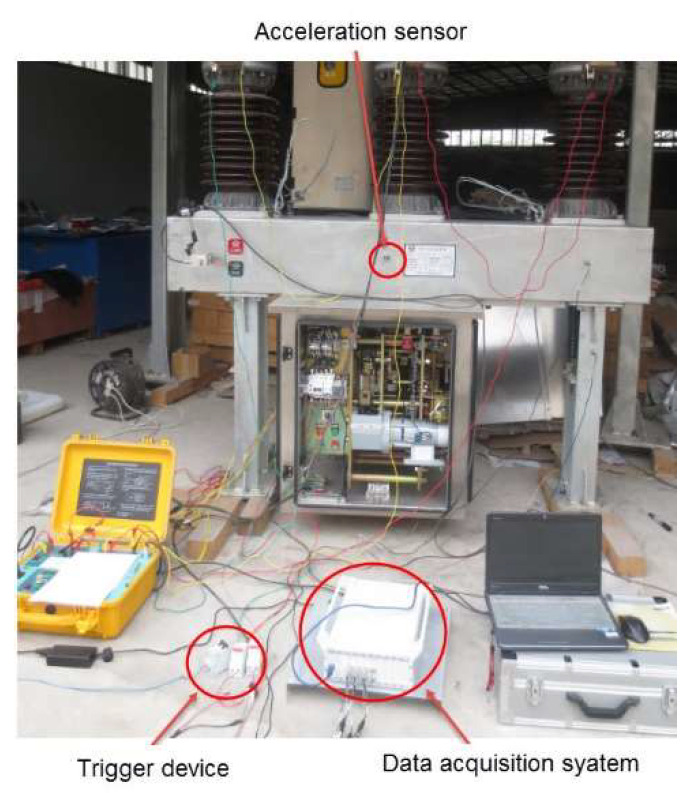
Experiment of high-voltage circuit breaker.

**Figure 3:**
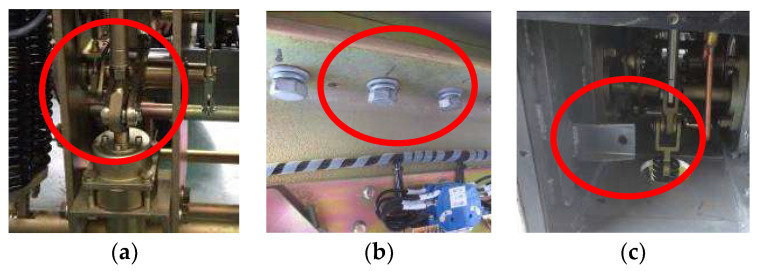
Simulative experiments of fault patterns. (**a**) Actuator fault; (**b**) Base screw looseness; (**c**) Buffer spring invalid.

We conducted an experiment on a 35 kV outdoor high-voltage SF_6_ circuit breaker, which is shown in Figure 2. The acceleration sensor was adopted to acquire the vibration signal in the experiment. Its performance parameters are as follows: manufacturer: Donghua Testing Technology Company; sensor type: DH131E; measuring range: 500 g; frequency response: 1–8000 Hz. After several tests at different positions, the base of the circuit breaker operating mechanism, in which the sensor was installed, was the best place to accurately obtain vibration signals of the operating mechanism. Some common faults often happen in the circuit breaker working process, including actuator fault, looseness of the base screw, and buffer spring invalidation. So, we simulated these faults in the laboratory. The length of the transmission rod was adjusted to simulate actuator fault, as shown in Figure 3a. The base screw was adjusted to simulate looseness of the base screw, as shown in Figure 3b. The buffer spring was removed to simulate buffer spring invalidation, as shown in Figure 3c. Fault simulation is shown in Figure 3.

**Figure 4:**
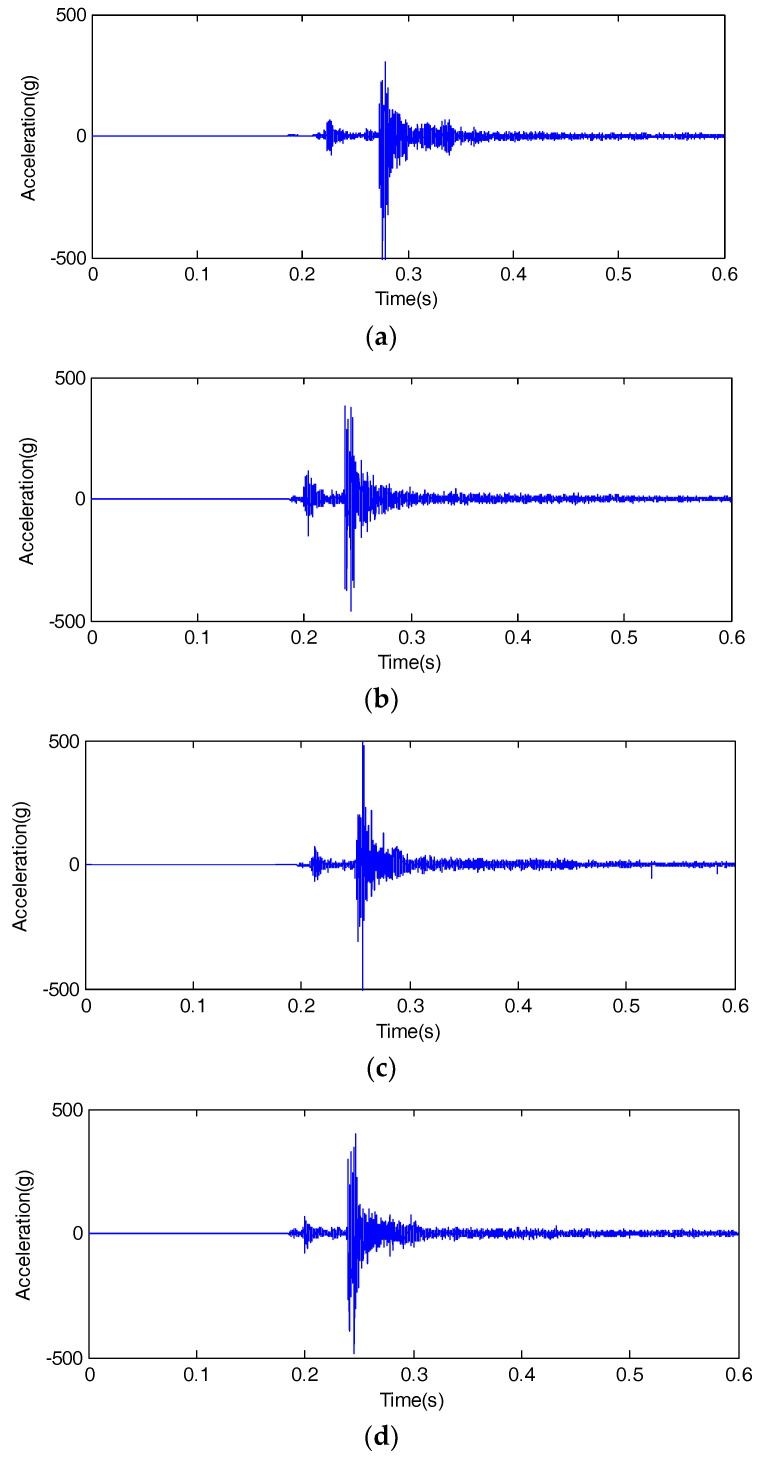
Vibration signal of high voltage circuit breaker. (**a**) Normal state; (**b**) Actuator fault; (**c**) Base screw looseness; (**d**) Buffer spring invalid.

Since only limited-length signals can be processed, the time domain signal needs to be truncated by multiplying a finite-width rectangular window. Trigger sampling was adopted in the data acquisition system. The trigger device is a power module that can transform the closing coil voltage to 5 V. In order to fully collect the vibration signal, we set a negative delay trigger. Data acquisition started 200 ms before the trigger. Considering the movement time of the spring operation mechanism, the acquisition time was designed with 600 ms. The sampling frequency was set as 10 kHz, and 2 input acquisition channels of the data acquisition system were used. We closed the circuit breaker 20 times in a normal state and 3 types of fault states, respectively. Six thousand data points were collected by the data acquisition system every time. Finally, we obtained 80 groups of data. After extracting the vibration data from the data acquisition system, we used MATLAB to preprocess the vibration data and calculate its average. After subtracting its mean value, the typical vibration signal in the closing moment is shown in Figure 4.

It can be seen from Figure 4 that there is no obvious difference or change when observing four kinds of vibration signals in the time domain. Therefore, it is necessary to adopt the appropriate method to extract fault features from vibration signals, then classification of the circuit breaker states is completed based on the feature vector. All of these can promote the development of vibration-signal analysis and fault-diagnosis methods.

### 5.2. Signal Processing

Due to the complex working environment of the circuit breaker, the collected vibration signals are mixed with electromagnetic factors and other noise. Therefore, it is necessary to eliminate the effect caused by noise.

**Figure 5:**
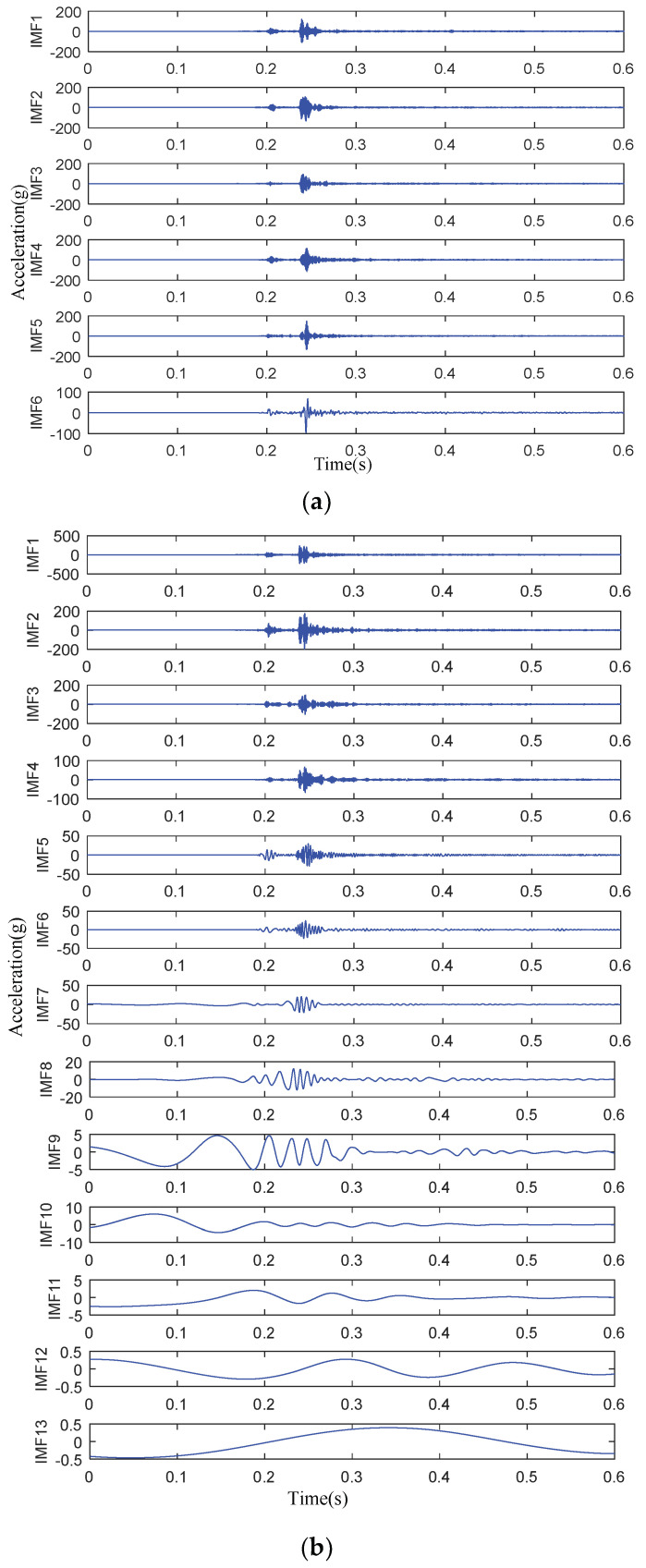
Decomposition results of different methods. (**a**) IMFs decomposed by VMD; (**b**) IMFs decomposed by EMD.

Taking the vibration signal of the actuator fault as an example, both EMD and VMD were used to process the vibration signal. The decomposition level of VMD is six levels and the result is shown in Figure 5.

It can be seen in Figure 5 that VMD decomposes the signal into six IMFs and each IMF waveform is consistent with the original vibration signal. On the other hand, EMD decomposes the signal into 13 IMFs. Not only are the number of iterations increased, but also the false mode appears in the IMFs, which means EMD is not beneficial to the extraction of useful IMFs. Therefore, VMD has excellent decomposition characteristics for processing nonperiodic vibration signals.

Calculate the correlation coefficient between IMFs and the original signal, which are the largest five IMFs selected to reconstruct the signal. This can eliminate the affect caused by noise.

### 5.3. Feature Vector Extraction and Analysis

**Figure 6:**
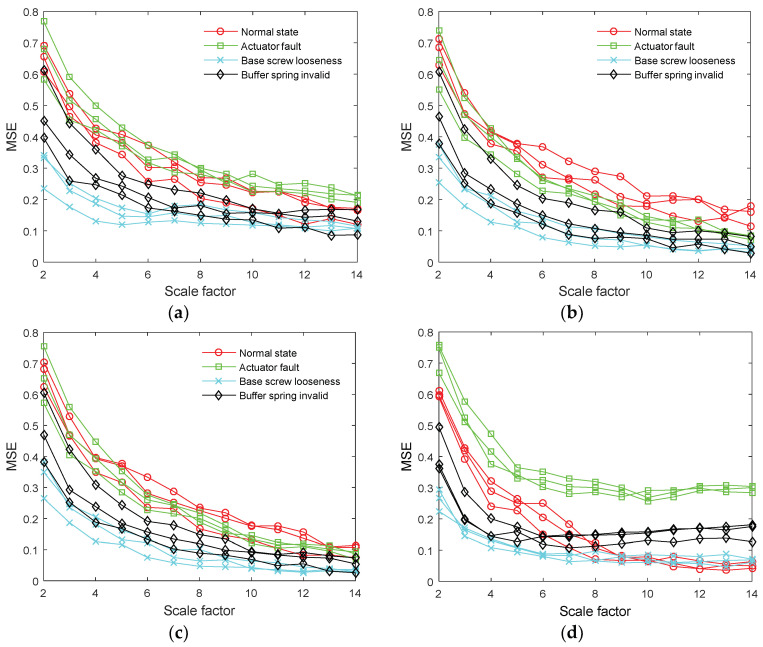
The extracted feature vector. (**a**) the feature vector of the original signal; (**b**) the feature vector extracted by EMD-MSE; (**c**) the feature vector extracted by EEMD-MSE; (**d**) is the feature vector extracted by VMD-MSE.

The multiscale entropy of the reconstructed signal was extracted as a feature vector, which can represent the change regulation of vibration data. In order to verify the effectiveness of VMD, both EMD and EEMD were used to process the vibration signal. Then, we reconstructed the signal by the above methods. This finally calculated multiscale entropy of the reconstructed signal. VMD-MSE, EMD-MSE, and EEMD-MSE were used to represent the feature vector extraction process. The extracted feature vectors are shown in Figure 6.

It can be seen from Figure 6a–c that the developing tendency of the feature vectors is consistent with each other. Under different scale factors, the values of multiscale entropy are crisscrossed and overlapped. It is difficult to distinguish the four states. Since useful information is not extracted, the fault feature is not obvious. Therefore, it is not ideal to process the vibration signal by EMD and EEMD. In Figure 6d, the change regulation of feature vectors is different from each other. Under different scale factors, not only the values of multiscale entropy but also the change of multiscale entropy is different. Comparing Figure 6d with Figure 6a, it indicates that VMD can extract useful information from the original signal. In addition, comparing Figure 6d with Figure 6b,c, it indicates that VMD can highlight the local characteristics of the data and shows better noise robustness than EMD and EEMD. Therefore, VMD-MSE is proven to be more powerful for dealing with the vibration signal and can effectively represent the state of the circuit breaker. 

Multiscale entropy, sample entropy, approximate entropy, and fuzzy entropy are usually used to extract the features of the vibration signal. They can represent the statistical features and meanwhile reflect the change regulation of the signal.

In comparison with different methods, both the multiscale entropy and the other methods are applied to the reconstructed signal. The sample entropy, approximate entropy, and fuzzy entropy of the signal are calculated, respectively, as indicated in Table 1.

On the theoretical side, sample entropy, approximate entropy, and fuzzy entropy can represent the changing regulation of data. Since their physical meanings are different, their values and changes are also different. The vibration signal of the circuit breaker is complicated and the vibration signal in the normal state and fault states are different, which lead to the results in Table 1, i.e., the entropy values of the vibration signal in different states are different.

In terms of classification, we had hoped the extracted fault feature has significant differences, which can help to improve the accuracy of classification. However, it is difficult to find some change rule in Table 1. When Table 1 is compared with Figure 6d, the multiscale entropy can show the change regulation of the vibration signal in different scale factors perfectly. Therefore, the feature vector constructed by multiscale entropy has significant differences. After comparing multiscale entropy with the other entropy methods, the advantage of multiscale entropy is verified.

### 5.4. Pattern Recognition and Classification

Due to the characteristics of the circuit breaker, the circuit breaker cannot open and close frequently, so we could only collect limited test data by experiment. Less sample data is not conducive to fault recognition training. The traditional neural network needs a large number of samples. The more samples there are, the more accurate the identification will be. SVM can complete recognition by less training samples, so it is more suitable for the circuit breaker than the other algorithms. We made use of the latest open-source 3.21 support vector machine in this paper by using the “one against the rest” strategy and built a support vector machine by combining normal samples with fault state samples. The final result was determined by the largest distance of the vector machine. 

The Kernel function is an important parameter to SVM due to the simple model and fewer parameters. The radial basis function was selected as the kernel function. Other than the proper kernel function, the penalty parameter *C* and the kernel function parameter *g* also can improve the performance of SVM. Parameter optimization was solved by using the grid search algorithm, which means we tried possible *C* and *g*. Then, the optimal penalty parameter *C* and kernel function parameter *g* were found via cross validation. The optimal penalty parameter *C* was 2 and kernel function parameter *g* was 16.

**Figure 7:**
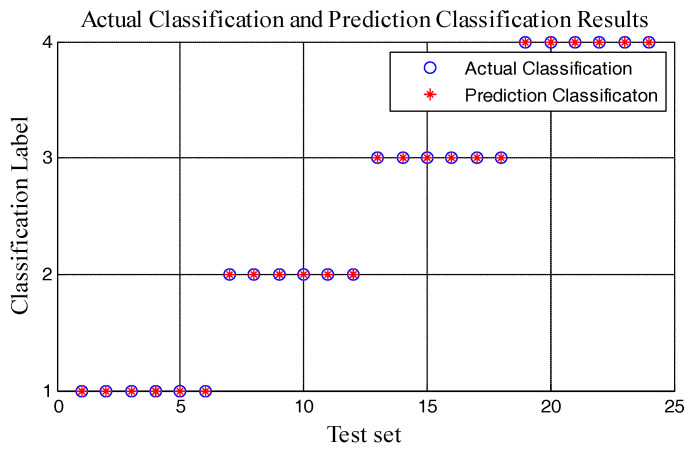
Comparison chart of actual classification and prediction classification.

In this experiment, the multiscale entropy of the 80 groups’ data was calculated. Then, the feature vector was completed. According to the feature vector, fault classification was realized. Fifty-six data groups were taken as the training set and the other 24 data groups were taken as a test set. Six groups of data were classified as a state and all of them did not overlap with the training set. After training SVM with the training set, the test set was classified. Classification results are shown in Figure 7.

Figure 7 shows that the classification of the test data is the same as the actual category. All test data is classified correctly. SVM can classify the state of the circuit breaker according to multiscale entropy and its change regulation. Therefore, the classification result based on multiscale entropy is excellent. Similarly, the feature vector is constructed by the rest methods and SVM is applied to classify the circuit breaker fault states. Classification results based on a different feature vector is shown in Table 2.

In order to verify the classification results of VMD-MSE, both the signal processing methods and entropy methods are taken into consideration. For the entropy methods, the multiscale entropy (MSE), sample entropy (SpEn), approximate entropy (ApEn), and fuzzy entropy (FuEn) are usually used to represent the statistical features of the signal. As such, VMD-SpEn, VMD-ApEn, VMD-FuEn, and VMD-MSE were used to serve as a feature vector and the classification results are shown in Table 2. Similarly, for the signal processing methods, both the original signal MSE, EMD-MSE, and EEMD-MSE were used to serve as the feature vector and the classification results are shown in Table 3.

As shown in Table 2, the classification result based on VMD-MSE is the best of all. This is because multiscale entropy can show the change regulation of the reconstructed signal in a different scale factor and the complexity of the signal can be reflected. The feature vector constructed by multiscale entropy is quite clear, so after training SVM with VMD-MSE, the classification accuracy of the test set is 100%. Since the feature vectors constructed by the other entropy methods are just single values, they have the feature of ambiguity. No matter which method is used as the input of SVM, the classification result of the corresponding feature vector is not ideal, i.e., the classification accuracy is low.

Comparing with different signal processing methods in Table 3, we found that the classification result of VMD-MSE is superior to the other signal processing methods. On the theoretical side, SVM can classify the circuit breaker’s states according to the change regulation and the difference of feature vectors, so the classification accuracy is related to the feature vector. The more different the feature vectors are, the more accurate the classification will be. Because there are few differences between EMD-MSE and EEMD-MSE, the classification results of EMD-MSE and EEMD-MSE are close to each other. Due to the change regulations of original signal MSE, EMD-MSE and EEMD-MSE are not obvious, and the results are of low classification accuracy. In contrast, the change regulation of VMD-MSE is obvious, so we can achieve excellent classification accuracy. Apart from EMD and EEMD, VMD can eliminate the signal noise effectively in order for the useful information to be extracted, which leads to the excellent classification result. 

In conclusion, VMD-MSE has a higher ability of extracting signal characteristics. The feature vector has obvious differences in different fault states, which means the proposed method has an excellent identification rate.

## 6. Conclusions

In this paper, the vibration signal during the closing process of a circuit breaker was analyzed. VMD was used to process the high voltage circuit breaker’s vibration signals and the reconstructed signal could eliminate the effect of noise. Afterward, the multiscale entropy of the reconstructed signal was calculated and selected as a feature vector. Lastly, after optimizing the SVM kernel-function parameters using the grid algorithm, the classification of the circuit breaker fault was completed using SVM. Results of this investigation are summarized below.

(1) The proposed method which combines the VMD multiscale entropy with SVM is first applied in vibration signal analysis of a high voltage circuit breaker. Characteristic information can be extracted from the original signal effectively. The proposed method can be applied to extract the fault characteristics and diagnose the circuit breaker’s fault types.
(2) Multiscale entropy can be used as a feature vector to characterize the circuit breaker’s states. This method is superior to the other methods. The multiscale entropy method is helpful for the circuit breaker’s state identification and fault classification.
(3) This research provided a practical solution for the fault diagnosis of a circuit breaker. The experimental results show that the proposed method can extract the feature vectors quickly and classify the state of the circuit breaker correctly.

## Figures and Tables

**Table 1:** Entropy of the reconstructed signal.

Fault States	Sample Entropy	Approximate Entropy	Fuzzy Entropy
Normal state	0.6311	1.5523	5.4207
Actuator fault	0.7381	0.6517	2.3090
Base screw looseness	0.3024	0.5632	7.0451
Buffer spring invalid	0.3674	0.6252	11.6892

**Table 2:** Classification of different entropy methods.

Serial Number	Different Entropy Methods	Classification Accuracy
1	VMD-SpEn	29.17%
2	VMD-ApEn	50%
3	VMD-FuEn	25%
4	VMD-MSE	100%

**Table 3:** Classification of different signal processing methods.

Serial Number	Different Signal Processing Methods	Classification Accuracy
1	Original signal MSE	33.33%
2	EMD-MSE	54.17%
3	EEMD-MSE	45.83%
4	VMD -MSE	100%
